# Effect of blood pressure control on the risk of proteinuria during bevacizumab treatment in patients with colorectal cancer: a single-center retrospective cohort study

**DOI:** 10.1186/s40780-024-00372-8

**Published:** 2024-08-23

**Authors:** Satoru Nihei, Junichi Asaka, Mizunori Yaegashi, Koichi Asahi, Kenzo Kudo

**Affiliations:** 1 Department of Pharmacy, Iwate Medical University Hospital, 2-1-1 Idaidori, Yahaba-cho, Shiwa-gun, Iwate, 028-3695 Japan; 2 Division of Clinical Pharmaceutics and Pharmacy Practice, Department of Clinical Pharmacy, School of Pharmacy, Iwate Medical University, 1-1-1 Idaidori, Yahaba-cho, Shiwa-gun, Iwate, 028-3694 Japan; 3 Department of Surgery, School of Medicine, Iwate Medical University, 1-1-1 Idaidori, Yahaba-cho, Shiwa-gun, Iwate, 028-3694 Japan; 4 Division of Nephrology and Hypertension, Department of Internal Medicine, School of Medicine, Iwate Medical University, 1-1-1 Idaidori, Yahaba-cho, Shiwa-gun, Iwate, 028-3694 Japan

**Keywords:** Bevacizumab, Proteinuria, Hypertension, Blood pressure (BP) control, Average systolic blood pressure (SBP), Colorectal cancer (CRC)

## Abstract

**Purpose:**

Pre-existing hypertension is reportedly a major risk factor for bevacizumab-induced proteinuria. However, few studies have focused on the effects of blood pressure (BP) control on proteinuria during bevacizumab treatment. We report a retrospective study of the association between poor BP control and the risk of developing proteinuria in patients with colorectal cancer (CRC).

**Methods:**

Data for CRC patients who received bevacizumab between April 2015 and March 2022 were retrospectively collected. Patients were categorized into two groups based on average systolic blood pressure (SBP) during treatment: normal SBP (< 140 mmHg) and high SBP (≥ 140 mmHg). To evaluate the association between average SBP and grade ≥ 2 proteinuria, we used a 3 month landmark analysis and a Cox regression model.

**Results:**

Of the 279 patients analyzed, 109 had high SBP and 170 had normal SBP. The cumulative incidence of grade ≥ 2 and severe proteinuria was significantly higher in the high compared to the normal SBP group (*p* < 0.001 and *p* = 0.028, respectively). Landmark analysis indicated significant differences in proteinuria between patients with and without high average SBP during the first 3 months of treatment (*p* = 0.002 and *p* = 0.015, respectively). Multivariate analysis showed that average SBP ≥ 140 mmHg was a significant independent risk factor for proteinuria (*p* = 0.008).

**Conclusion:**

Landmark analysis showed that BP status during the first 3 months of bevacizumab treatment influences the risk of subsequent proteinuria. Therefore, timely diagnosis and stricter BP control are recommended for at least the first 3 months to avoid severe proteinuria.

**Supplementary Information:**

The online version contains supplementary material available at 10.1186/s40780-024-00372-8.

## Introduction

Bevacizumab is a recombinant humanized monoclonal antibody that targets vascular endothelial growth factor (VEGF), inhibiting its binding to its receptors. The antitumor efficacy of bevacizumab relies on inhibition of the VEGF-signaling pathway, which is involved in endothelial survival, vascular permeability, and, therefore, tumor angiogenesis [1]. Bevacizumab, the first anti-VEGF agent approved for combination use with chemotherapy, has been shown to prolong survival in patients with unresectable advanced or recurrent colorectal cancer (CRC) [2].

The most commonly reported side effects of bevacizumab are hypertension and proteinuria [3], with incidence rates of 21–62% and 3–43%, respectively [4–7]. The hypertension and proteinuria are occasionally severe enough to be life threatening [8, 9]. While the observed hypertension can generally be managed with antihypertensive (aHT) agents, such as renin-angiotensin system inhibitors (RASIs), including angiotensin-converting enzyme inhibitors (ACEIs) and angiotensin receptor blockers (ARBs), and dihydropyridine calcium channel blockers (CCBs), proteinuria is often treatment limiting, and necessitates bevacizumab dose interruption or dose reduction [10]. Thus, the management of proteinuria in patients treated with bevacizumab is important for maximizing the therapeutic effects of the drug.

Several mechanisms are assumed to be involved in the development of hypertension with bevacizumab treatment, including a decrease in nitric oxide and prostaglandin I2 production, rarefaction of blood vessels, vascular stiffness and disturbed endothelin function, although the exact mechanism has not yet been clarified [11]. Proteinuria might be caused by direct inhibition of the effects of VEGF on glomerular endothelial function, or indirectly through the increase in blood pressure, but is most probably due to a combination of both factors [12–14].

Previous studies suggested that pre-existing hypertension is a major risk factor for bevacizumab-induced proteinuria [15–17]. Although blood pressure (BP) control plays a major role in the development of proteinuria in patients with other kidney diseases, including diabetic and nondiabetic kidney diseases [18–20], few studies have focused on the effects of BP control on proteinuria in patients receiving bevacizumab. Therefore, this retrospective study focused on the effects of poor BP control during bevacizumab treatment on the risk of developing proteinuria in CRC patients.

## Materials and methods

The study was approved by the Medical Ethics Committee of Iwate Medical University (MH2021-106). All procedures were conducted in accordance with the Declaration of Helsinki and its amendments. The institutional review board waived the requirement for written informed consent due to the retrospective nature of this study. All patients were provided an opportunity to opt out of the study.

### Study design and participants

Patients with CRC who underwent bevacizumab treatment for the first time at Iwate Medical University Hospital (Iwate, Japan) between April 2015 and March 2022 were reviewed. Data for all patients were retrospectively collected from their electronic medical records. The data cutoff date was June 2023.

The inclusion criteria were: age ≥ 18 years; diagnosis of unresectable advanced and/or recurrent CRC; and administration of bevacizumab combined with chemotherapy, with completion of at least one cycle of treatment. The exclusion criteria were: prior treatment with anti-VEGF agents at baseline before commencement of bevacizumab treatment; pre-existing proteinuria; baseline estimated glomerular filtration rate (eGFR) < 30 mL/min/1.73 m^2^; history of diabetes, dyslipidemia, cardiovascular disease and stroke; and incomplete clinical data.

Bevacizumab at a dose of either 5 mg/kg every 2 weeks or 7.5 mg/kg every 3 weeks, in combination with chemotherapy, was administered intravenously. Treatment was continued until tumor progression or development of unmanageable adverse events.

For all patients, BP was measured in the sitting position using automatic BP monitors (TM-2657, A&D Company, Ltd., Tokyo, Japan) by nursing staff once they arrived at the clinic but before they saw the doctor. In this study, morning BP readings measured at baseline and every 2–3 weeks were assessed. Baseline BP was the value measured on the day of bevacizumab treatment initiation, or within 30 days prior to treatment initiation if the BP reading of the same day was not available. The presence of hypertension in patients was assessed by a recorded prescription for aHT agents. Average systolic and diastolic blood pressures (SBP and DBP) during treatment were calculated as: average SBP/DBP = sum of the values of SBP/DBP measured before every treatment divided by the number of treatments [21]. Patients were categorized into two groups based on average SBP: normal SBP (< 140 mmHg) and high SBP (≥ 140 mmHg). The target SBP value of < 140 mmHg set in our study might be reasonable, especially for patients who are unable to either tolerate or achieve stricter BP control due to ongoing chemotherapy [22].

### Definitions and study outcomes

The primary outcome of this study was development of grade ≥ 2 proteinuria, defined as a urine protein-creatinine ratio (UPCR) of ≥ 1.0 g/gCr, according to the Common Terminology Criteria for Adverse Events version 5.0 (CTCAE v5.0). Proteinuria grade was determined based on the results of UPCR instead of 24-hour urine protein according to the CTCAE v5.0 [23]. The secondary outcome was an event of severe proteinuria, defined as a UPCR of ≥ 2.0 g/gCr. In cases with UPCR ≥ 2.0 g/gCr, it is advised that bevacizumab treatment be withheld until UPCR decreases to < 2.0 g/gCr [3].

### Statistical analysis

Categorical and numerical variables are reported as the number (percentage, %) and median (interquartile range, 25th-75th percentile), respectively. The χ^2^-test, Fisher’s exact test, and Mann–Whitney U-test were used as appropriate. The cumulative incidence of grade ≥ 2 and severe proteinuria was estimated using Kaplan–Meier curve analysis in each group, and groups were compared using the log-rank test.

Taking into account the lead-time bias due to the time-dependent nature of hypertension, we performed a 3 month landmark analysis. Three months was chosen as the landmark time point because hypertension screening, treatment and control were expected to be achieved within 3 months after the onset of treatment [24–26]. This landmark analysis had two purposes. The first was to adjust for possible bias arising from falsely categorizing patients in the normal SBP (< 140 mmHg) group if they had died or stopped taking bevacizumab before experiencing the side effect of hypertension. In addition, this analysis also adjusted for the possibility of including patients who developed spurious hypertension long after bevacizumab exposure, which likely is not true bevacizumab-induced hypertension. The second purpose was to adjust for possible guarantee-time bias introduced by a spurious survival advantage, because, by design, the patients categorized in the high SBP (≥ 140 mmHg) group had to survive till their mean SBP reached ≥ 140 mmHg in order to be assigned as cases. Accordingly, only patients who did not develop proteinuria events or require discontinuation of bevacizumab at this point were included in our analysis, and were divided into patients who did and did not experience an average SBP of ≥ 140 mmHg during the first 3 months of treatment.

Univariate and multivariate Cox regression analyses were performed to identify independent risk factors for proteinuria. To adjust for the potential effects of the covariates, univariate Cox regression analyses were initially performed to assess the association between the onset of grade > 2 proteinuria and each candidate variable, including sex, age, body mass index (BMI), eGFR, bevacizumab dose, pre-existing hypertension, baseline SBP/DBP, and average SBP/DBP. Cutoffs for age, BMI, eGFR, SBP and DBP were considered based on international guidelines or previous recommendations [27–29]. The variables found in univariate analysis to have a p value of < 0.20 were entered into the multivariable model using forward stepwise analysis. The results are given as hazard ratios (HR) with 95% confidence intervals (CI). A p value of < 0.05 was considered to be statistically significant. Data were analyzed using IBM SPSS Statistics for Windows, version 27.0.

## Results

### Baseline characteristics

A total of 353 patients were screened for eligibility; 74 were excluded for the following reasons: prior treatment with other anti-VEGF agents at baseline before bevacizumab treatment (*n* = 4), pre-existing proteinuria (*n* = 22), baseline eGFR < 30 mL/min/1.73 m^2^ (*n* = 9), history of diabetes, dyslipidemia, cardiovascular diseases and stroke (*n* = 31), and incomplete clinical data (*n* = 12), with overlap. Then, a total of 279 patients were analyzed in this study, 109 with average SBP ≥ 140 mmHg and 170 with average SBP < 140 mmHg for the full period. Table 1 presents the baseline characteristics of the entire patient dataset stratified by average SBP status. The high SBP group was more likely to have higher BMI, SBP, DBP, and presence of hypertension than the normal SBP group. There were no significant differences in age, sex, serum creatinine level, eGFR, bevacizumab dose, and the chemotherapy combination between the two groups. A total of 89 patients (32%) were receiving aHT agents at baseline. These agents included RASIs, CCBs, diuretics, and α/β- or β-blockers (Supplementary Table 1).

**Table 1:** Baseline characteristics

Clinical parameter	Normal SBP (*n* = 170)	High SBP (*n* = 109)	*p* value
Age, years	64 (56–70)	65 (59–71)	0.453
Sex			0.459
Male	89 (52)	62 (57)	
Female	81 (48)	47 (43)	
BMI, kg/m^2^	22 (20–24)	23 (21–25)	0.031
Serum creatinine level, mg/dL	0.67 (0.55–0.81)	0.69 (0.57–0.80)	0.569
eGFR, mL/min/1.73 m^2^	79 (63–100)	80 (63–103)	0.501
SBP	116 (110–122)	133 (129–140)	< 0.001
DBP	67 (62–70)	79 (74–85)	< 0.001
Distribution of baseline antihypertensive agents	36 (21)	53 (49)	< 0.001
RASIs	17 (10)	31 (28)	< 0.001
CCBs	26 (15)	30 (28)	0.013
Diuretics	5 (3)	8 (7)	0.089
α/β- or β-blockers	1 (1)	2 (2)	0838
Bevacizumab dose			0.937
7.5 mg/kg, 3 weeks	71 (42)	45 (41)	
5 mg/kg, 2 weeks	99 (58)	64 (59)	
Combination chemotherapy			0.362
Oxaliplatin-based chemotherapy	152 (89)	101 (93)	
Irinotecan-based chemotherapy	18 (11)	8 (7)	

Values are presented as the n (%) or median (interquartile range [25th-75th percentile]).

Abbreviations: SBP: systolic blood pressure; DBP: diastolic blood pressure; BMI: body mass index; eGFR: estimated glomerular filtration rate; RASIs: renin-angiotensin system inhibitors; CCBs: calcium channel blockers

### Blood pressure trends after starting bevacizumab

**Fig. 1:**
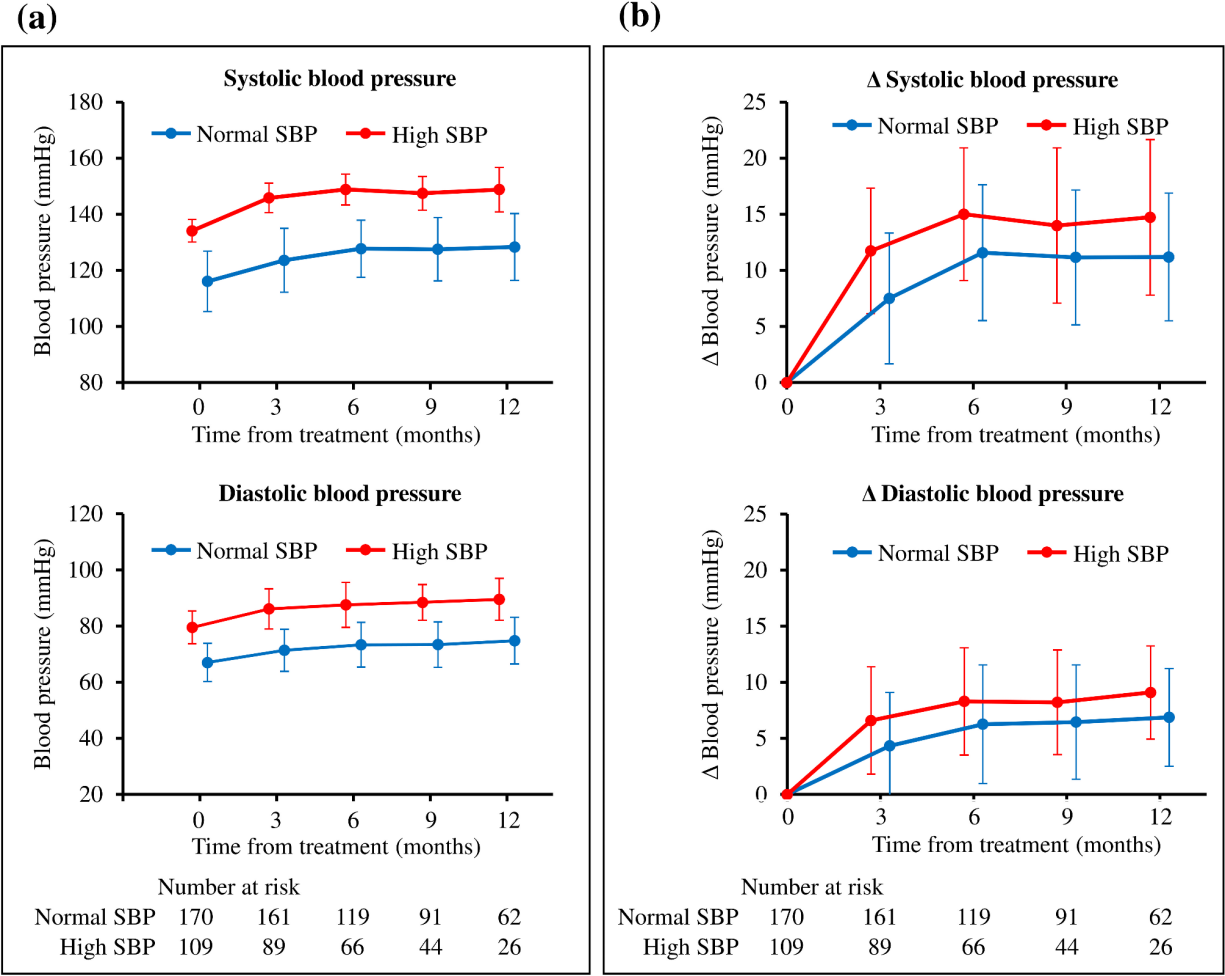
Comparisons of blood pressure trends during bevacizumab administration in normal and high SBP groups. (**a**) Blood pressure trends in each group. (**b**) Changes in blood pressure from baseline in each group. Data are expressed as the mean and standard deviation. Abbreviations: SBP: systolic blood pressure

Trends in SBP and DBP differed between the two groups, being consistently higher in the high SBP group than the normal SBP group (Fig. 1a). Both normal SBP and high SBP groups showed an increasing trend for SBP and DBP from 1 month after the start of bevacizumab (+ 7.5 and + 11.7 mmHg, respectively), and a leveling off trend after 3 months (+ 11.6 and + 15.0 mmHg, respectively) (Fig. 1b). None of the patients required interruption of bevacizumab treatment due to hypertension during the follow-up period.

### Cumulative incidence of grade ≥ 2 and severe proteinuria

**Fig. 2:**
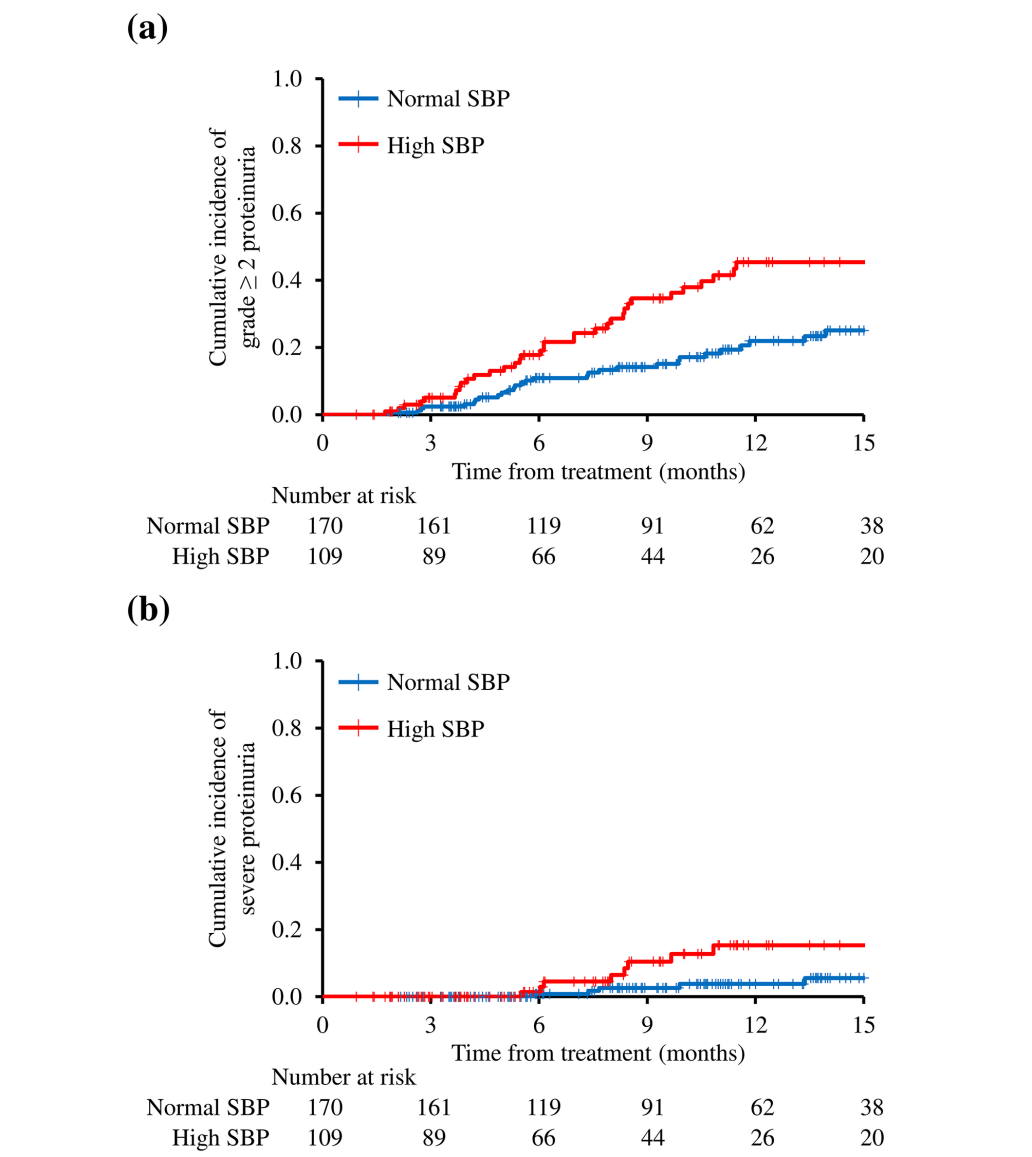
Kaplan-Meier estimates of the cumulative incidence of grade ≥ 2 and severe proteinuria. (**a**) Grade ≥ 2 proteinuria in normal SBP and high SBP groups (*p* < 0.001). (**b**) Severe proteinuria in normal SBP and high SBP groups (*p* = 0.028). Abbreviations: SBP: systolic blood pressure

Based on the data cutoff date, the median follow-up period was 8.6 (interquartile range 4.8–13.7) months. The median number of bevacizumab cycles administered was 14 (interquartile range, 8–23 cycles). During the follow-up period, 63 patients (23%) developed grade ≥ 2 proteinuria and 13 patients (5%) had severe proteinuria. Kaplan-Meier curves for grade ≥ 2 or severe proteinuria up to 12 months after starting bevacizumab in the full dataset of 279 patients are shown in Fig. 2. The cumulative incidence of grade ≥ 2 proteinuria at 12 months was 45.9% (95% CI: 34.1–57.8) in the high SBP group and 21.5% (95% CI: 13.8–29.1%) in the normal SBP group (*p* < 0.001) (Fig. 2a). The cumulative incidence of severe proteinuria was 3.8% (95% CI: 0.1–7.5) in the high SBP group and 15.3% (95% CI: 5.3–25.4) in the normal SBP group (*p* = 0.028) (Fig. 2b).

Univariate and multivariate Cox regression analyses were performed to determine independent risk factors for grade ≥ 2 proteinuria (Table 2). Univariate analysis showed that BMI ≥ 25 kg/m^2^ (HR 1.79, 95% CI 1.01–3.15, *p* = 0.045), pre-existing hypertension (HR 1.86, 95% CI 1.13–3.05, *p* = 0.015) and average SBP ≥ 140 mmHg during treatment (HR 2.27, 95% CI 1.38–3.73, *p* = 0.001) were related to the development of grade ≥ 2 proteinuria. Variables with p values < 0.20 were included in multivariate analysis followed by backward stepwise regression analysis. Finally, multivariate analysis showed that average SBP ≥ 140 mmHg during treatment was a significant independent risk factor for proteinuria (HR 1.98, 95% CI 1.18–3.33, *p* = 0.010).

**Table 2:** Univariate and multivariate Cox regression analyses of risk factors for grade ≥ 2 proteinuria

Variables	Univariate			Multivariate		
	HR	95% CI	*p* value	HR	95% CI	*p* value
Sex, male	1.04	0.63–1.71	0.871			
Age ≥ 65 years	1.19	0.72–1.94	0.501			
BMI ≥ 25 kg/m^2^	1.79	1.01–3.15	0.045	1.50	0.84–2.68	0.172
eGFR < 60 mL/min/1.73 m^2^	1.26	0.70–2.25	0.437			
Bevacizumab dose of 7.5 mg/kg	1.28	0.78–2.10	0.327			
Pre-existing hypertension	1.86	1.13–3.05	0.015	1.43	0.84–2.43	0.185
Baseline SBP ≥ 140 mmHg	1.73	0.97–3.09	0.065			
Baseline DBP ≥ 90 mmHg	1.88	0.93–3.81	0.080			
Average SBP ≥ 140 mmHg during treatment	2.27	1.38–3.73	0.001	1.98	1.18–3.33	0.010
Average DBP ≥ 90 mmHg during treatment	1.72	0.93–3.16	0.082			

Abbreviations: HR: hazard ratio; CI: confidence interval; BMI: body mass index; eGFR: estimated glomerular filtration rate; SBP: systolic blood pressure; DBP: diastolic blood pressure

### Landmark analysis

**Fig. 3:**
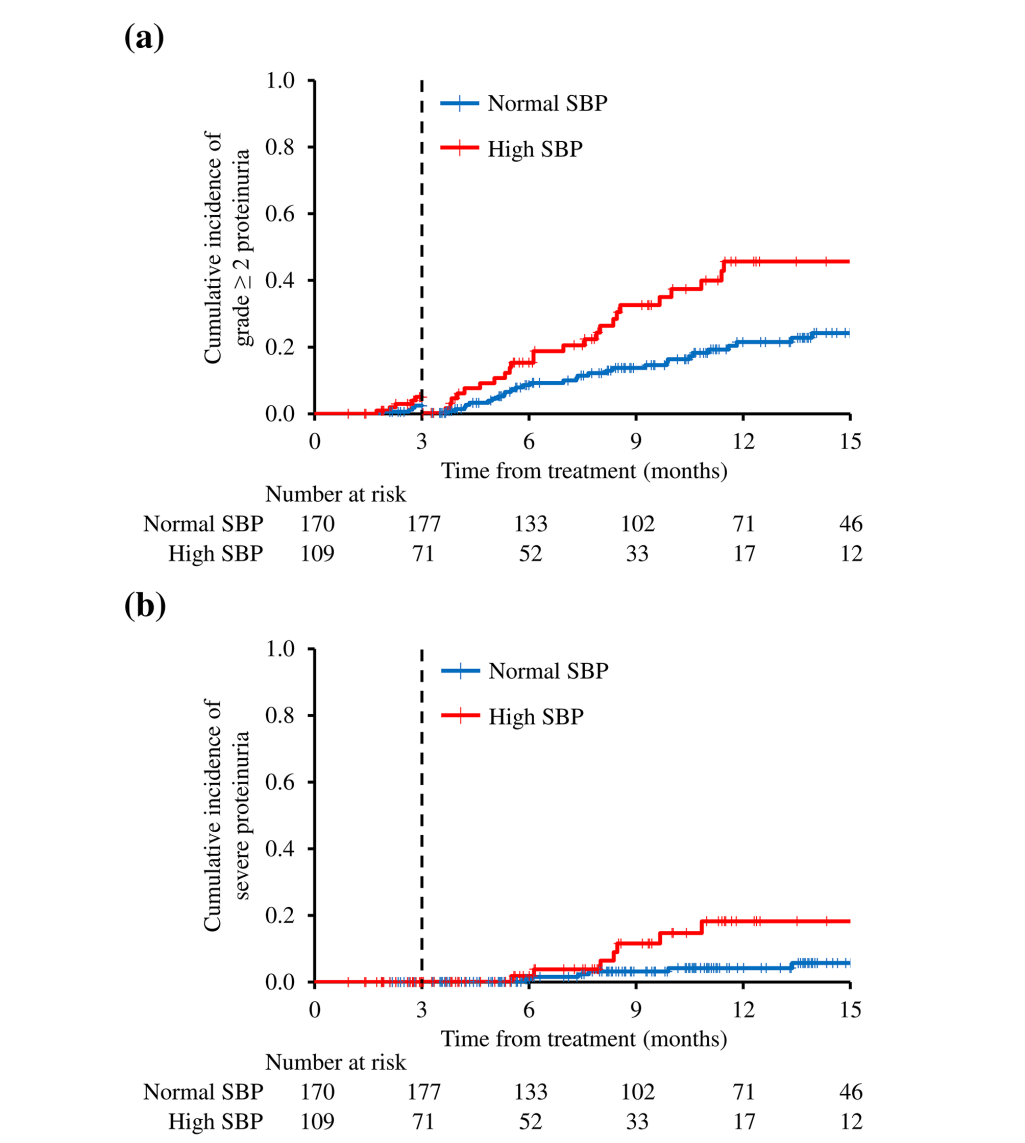
Landmark analysis performed using each patient’s average SBP status at 3 months. The cumulative incidence was calculated from the 3 month landmark time point. (**a**) Grade ≥ 2 proteinuria in normal SBP and high SBP groups before and after the landmark time point (*p* = 0.244 and *p* = 0.002, respectively). (**b**) Severe proteinuria in normal SBP and high SBP groups before and after the landmark time point (*p* = 1.000 and *p* = 0.015, respectively). Abbreviations: SBP, systolic blood pressure

In this landmark analysis, in which each patient’s average SBP status was determined at 3 months, nine patients who had grade ≥ 2 proteinuria and 22 patients who stopped bevacizumab before this point were excluded. The remaining 248 patients (88.9%) were included in this analysis, 71 in the high SBP group and 177 in the normal SBP group. After the landmark time point, the cumulative incidence of grade ≥ 2 proteinuria at 12 months was 37.4% (95% CI: 25.4–49.4) in the high SBP group and 15.0% (95% CI: 9.3–20.7%) in the normal SBP group (*p* = 0.002) (Fig. 3a), and the cumulative incidence of severe proteinuria was 3.8% (95% CI: 0.1–7.5) in the high SBP group and 15.3% (95% CI: 5.3–25.4) in the normal SBP group (*p* = 0.015) (Fig. 3b). Before the landmark time point, the cumulative incidences of grade ≥ 2 or severe proteinuria were not significantly different between the two groups (*p* = 0.244 and *p* = 1.000, respectively).

Univariate and multivariate Cox regression analyses after the landmark time point were performed to determine independent risk factors for grade ≥ 2 proteinuria (Table 3). Univariate analysis showed that pre-existing hypertension (HR 1.86, 95% CI 1.09–3.19, *p* = 0.023) and average SBP ≥ 140 mmHg within 3 months (HR 2.33, 95% CI 1.36–4.00, *p* = 0.002) were related to the development of grade ≥ 2 proteinuria. Variables with p values < 0.20 were included in multivariate analysis followed by backward stepwise regression analysis. Finally, multivariate analysis showed that average SBP ≥ 140 mmHg within 3 months was a significant independent risk factor for proteinuria (HR 2.11, 95% CI 1.21–3.67, *p* = 0.008).

**Table 3:** Univariate and multivariate Cox regression analyses of risk factors for grade ≥ 2 proteinuria after the landmark time point

Variables	Univariate			Multivariate		
	HR	95% CI	*p* value	HR	95% CI	*p* value
Sex, male	0.83	0.49–1.42	0.497			
Age ≥ 65 years	1.27	0.74–2.17	0.379			
BMI ≥ 25 mg/kg^2^	1.51	0.79–2.86	0.212			
eGFR < 60 mL/min/1.73 m^2^	1.44	0.78–2.64	0.243			
Bevacizumab dose of 7.5 mg/kg	1.22	0.71–2.08	0.473			
Pre-existing hypertension	1.86	1.09–3.19	0.023	1.60	0.92–2.77	0.096
Baseline SBP ≥ 140 mmHg	1.75	0.94–3.27	0.079			
Baseline DBP ≥ 90 mmHg	1.94	0.92–4.12	0.083			
Average SBP ≥ 140 mmHg within 3 months	2.33	1.36–4.00	0.002	2.11	1.21–3.67	0.008
Average DBP ≥ 90 mmHg within 3 months	1.79	0.90–3.56	0.097			

Abbreviations: HR: hazard ratio; CI: confidence interval; BMI: body mass index; eGFR: estimated glomerular filtration rate; SBP: systolic blood pressure; DBP: diastolic blood pressure

### Effects of antihypertensive agent classes on proteinuria

**Fig. 4:**
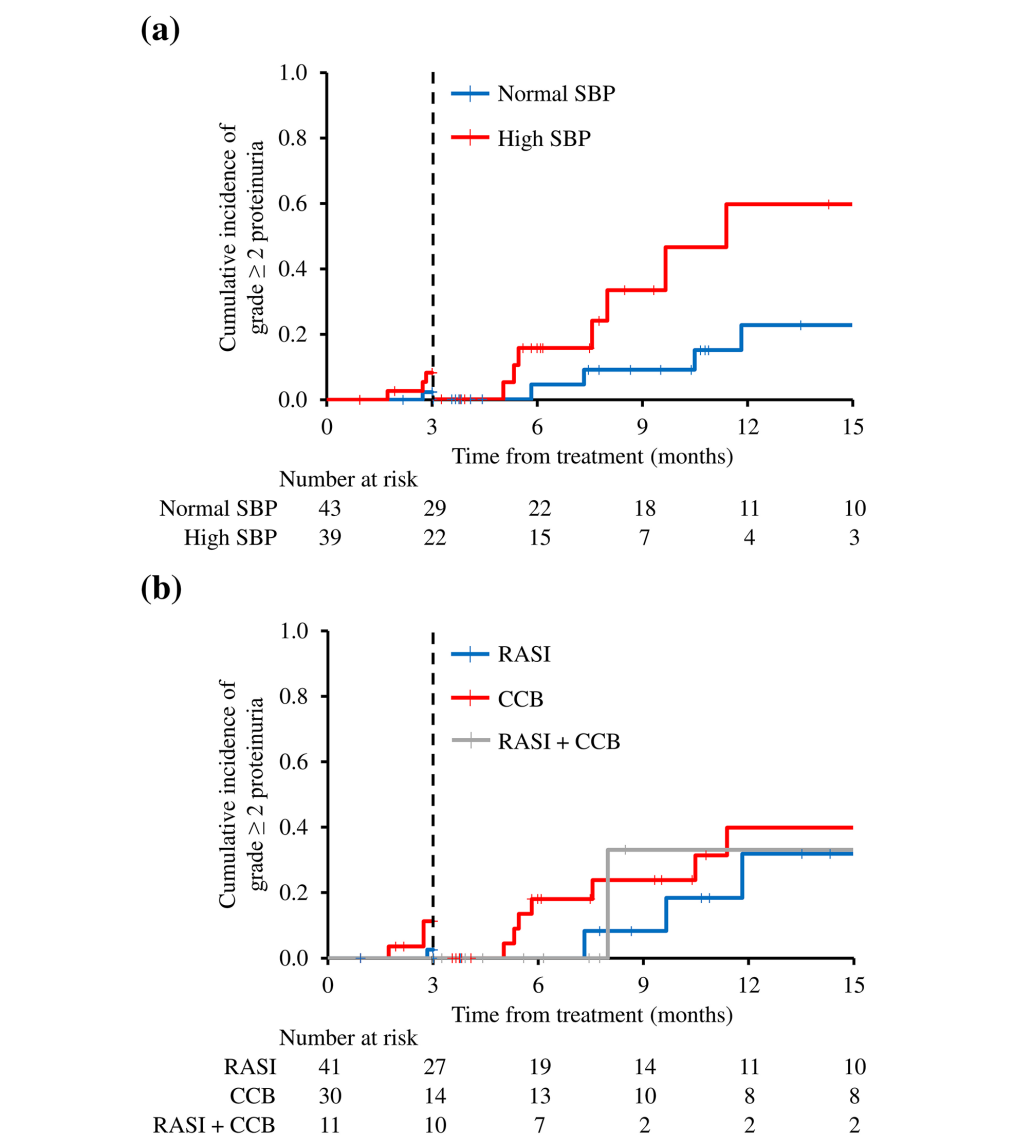
Subgroup analysis of patients who received antihypertensive treatment during bevacizumab treatment using 3 months as the landmark. The cumulative incidence was calculated from the 3 month landmark time point. (**a**) Grade ≥ 2 proteinuria in normal SBP and high SBP groups before and after the landmark time point (*p* = 0.238 and *p* = 0.034, respectively). (**b**) Grade ≥ 2 proteinuria based on antihypertensive agent classes before and after the landmark time point (*p* = 0.336 and *p* = 0.708, respectively). Abbreviations: SBP, systolic blood pressure; RASI, renin-angiotensin system inhibitor; CCB, calcium channel blocker

An aHT treatment was initiated in 82 (29%) of the 279 patients. This included new aHT treatment (*n* = 66) or intensification of previously prescribed aHT treatment (*n* = 16). Given the reported effect of bevacizumab in increasing BP, we analyzed if the high BP levels over time have an influence on proteinuria in patients who receive aHT treatment during bevacizumab treatment, i.e., patients with new onset or worsening of hypertension. Using the landmark model, the incidence of grade ≥ 2 proteinuria was compared between normal SBP (*n* = 29) and high SBP (*n* = 22) groups who initiated aHT treatment during the first 3 months of treatment. The results of this subgroup analysis after the landmark time point showed that the cumulative incidence of grade ≥ 2 proteinuria at 12 months was 60.2% (95% CI: 27.9–92.6) in the high SBP group and 22.9% (95% CI: 2.4–43.4%) in the normal SBP group (*p* = 0.034) (Fig. 4a). Before the landmark point, the cumulative incidences of grade ≥ 2 proteinuria were not significantly different between the two groups (*p* = 0.238).

RASIs were the most frequently prescribed agents for BP increase during bevacizumab treatment (*n* = 52), followed by CCB (*n* = 41), diuretics (*n* = 6), and α/β- or β-blockers (*n* = 1) (Supplementary Table 2). In 11 patients, ≥ 2 classes of aHT agents were prescribed. Given the low prescription numbers of the other aHT agents, the effect of aHT agents on proteinuria was only analyzed in patients with RASI and/or CCB. Landmark analyses were performed as before, dividing the patients into three groups according to the aHT agents started during the first 3 months of treatment: RASI, CCB, and RASI + CCB. However, there was no difference in the cumulative incidence of grade ≥ 2 proteinuria between the three groups both before and after the landmark point (*p* = 0.336 and *p* = 0.708, respectively), suggesting no advantage of any one drug over others in the prevention of proteinuria (RASI vs. CCB: *p* = 0.816; RASI vs. RASI + CCB: *p* = 0.914; CCB vs. RASI + CCB: *p* = 0.917) (Fig. 4b).

## Discussion

The aim of this retrospective study was to evaluate the association between poor BP control and the risk of developing proteinuria in unresectable advanced and/or recurrent CRC patients treated with bevacizumab using both landmark analysis and a Cox model. Our results showed that the incidence of bevacizumab-induced proteinuria was significantly higher in patients with high average SBP during treatment. The clinical significance of these findings is noteworthy, as the association remained significant even in severe proteinuria cases (UPCR ≥ 2 g/gCr). This is the first study to show an association between high BP levels over time and increased proteinuria during bevacizumab treatment in patients with unresectable advanced or recurrent CRC.

Existing recommendations for the management of anti-VEGF agent-induced proteinuria are based on expert opinion and not on robust evidence, resulting in inadequate proteinuria control in patients receiving bevacizumab. Some recommendations suggest that patients with proteinuria should receive appropriate BP management using antihypertensive treatment (if hypertensive), and the initial agents of choice are either ACEIs or ARBs [30]. The bevacizumab package insert recommends monitoring for the development of proteinuria, but does not provide specific recommendations, except for temporary suspension or discontinuation of bevacizumab treatment in patients with moderate to severe proteinuria [3]. The findings of this study provide insights into the role of hypertension during bevacizumab treatment in exacerbating the proteinuria, which should help healthcare providers better understand the optimal management of anti-VEGF agent-induced proteinuria in clinical practice.

VEGF and its receptors play an important role in vascular homeostasis and are widely expressed in the body. It has been theorized that inhibitors of VEGF or the signaling of VEGF receptors (VEGFRs) induce hypertension via decreased production of vasodilators (nitric oxide, prostacyclin), increased production of vasoconstrictors (endothelin-1), and rarefaction of microvascular endothelial cells [11]. This might lead to renal hemodynamic compromise and subsequent proteinuria. Numerous studies have suggested that hypertension is associated with the onset of proteinuria in patients with various types of cancers treated with bevacizumab, with this association receiving further support from several studies evaluating patients treated with other inhibitors targeting VEGF signaling, including monoclonal antibodies targeting VEGF and small molecules targeting VEGFR [15, 31]. We previously reported that baseline SBP/DBP ≥ 140/90 mmHg was a significant risk factor for the onset of proteinuria in non-small cell lung cancer patients receiving bevacizumab combined with chemotherapy [17]. Of note, a subgroup analysis in one study suggested that high average SBP during bevacizumab treatment was significantly associated with severe proteinuria, consistent with our findings [21]. Thus, the results of these studies likely support the hypothesis that proteinuria might result from increased intraglomerular pressure secondary to arterial hypertension. However, in a preclinical study, the variable response of hypertension and proteinuria to different aHT agents suggests that these side effects are, at least in part, unrelated [32]. We addressed this issue by performing detailed analysis taking these factors into consideration. Subgroup analysis including only patients with new-onset or worsening hypertension during bevacizumab treatment showed that high average SBP significantly increases proteinuria. This implicated average SBP over time, rather than the onset of hypertension per se, as a key determinant of the positive association between SBP and proteinuria. However, some cases also documented proteinuria in the absence of hypertension, indicating that it is not the only trigger for bevacizumab-induced proteinuria.

Although there might be an association between high BP levels over time and proteinuria, it is not yet clear whether one is secondary to the other, or vice versa, or if both are independently caused by VEGF inhibition. So far, the possibility that proteinuria and hypertension are associated is based on the observation that patients who develop proteinuria are more likely to become hypertensive [21, 33]. Moreover, some previous results should be interpreted cautiously because they do not deal appropriately with the guarantee-time bias. Our findings were all based on a 3 month landmark analysis, thereby reducing the possible bias due to reverse causation, in which the proteinuria occurs before hypertension, rendering our results robust. Previous reports showed that the time to the onset of hypertension upon receiving bevacizumab varies, but is frequently observed early (at 1–3 months) [24–26, 34]. Likewise, our findings showed that blood pressure increases at 1 month after the start of bevacizumab, peaking at approximately 3 months. On the other hand, most cases of grade ≥ 2 proteinuria were observed 3 months after commencing treatment (median 5.3 months). These findings are consistent with previous observations regarding bevacizumab-induced proteinuria; in several studies, the median onset time of high-grade proteinuria was 5.6 months [8]. Landmark analysis revealed that grade ≥ 2 or severe proteinuria in patients with high average SBP was significantly greater after 3 months, and not within 3 months, of bevacizumab treatment commencement. This result indicates that BP status during the first 3 months of treatment influences the risk of subsequent proteinuria. In addition, multivariable analysis considering pre-existing hypertension showed that a higher average SBP was the only risk factor related to bevacizumab-induced proteinuria, highlighting the need for appropriate BP control during treatment.

In diabetic and non-diabetic chronic nephropathies, high BP is a major determinant of disease progression, and BP reduction is renoprotective [35]. Reducing blood pressure with RASI treatment has the additional benefit of lowering glomerular hypertension, which, in turn, optimizes glomerular-sieving properties [36, 37]. In chronic nephropathies with proteinuria, RASI treatment has been shown to decrease proteinuria [38–40]. However, the data are inconclusive regarding whether the benefit extends to patients without proteinuria. In a preclinical study of sunitinib treatment in rats, ACEIs prevented anti-VEGF agent-related proteinuria [11, 41]. Although some retrospective studies have suggested a beneficial effect of RASIs on proteinuria [17, 42], the absence of randomized controlled trials proving the superiority of RASIs over other aHT agents in patients who develop proteinuria while on anti-VEGF agent treatment must be considered. In our study, use of RASIs or CCBs for hypertension during treatment was common, with no difference in the development of proteinuria between the two. Subgroup analysis according to class of aHT agents failed to differentiate between the effect of ACEIs or ARBs, and CCB subtypes, including L-type, L/T-type or L/N-type. Although intrarenal effects may differ between classes and between individual drugs within certain classes, further investigation is required to verify these findings. Therefore, aHT agents should be tailored according to the patient’s comorbidities, as in noncancer patients.

This study has several key limitations. First, the data for aHT agent usage, including the exact change in drug dosage and patient adherence to therapy, which might affect proteinuria, were incomplete. In addition, we did not analyze temporal trends in changes in average SBP or their associations with the development of proteinuria. Second, owing to the retrospective nature of our analysis, there were unavoidable selection biases and confounders. We did not perform propensity score matching due to the small cohort of patients with higher average SBP. Third, since patients with pre-existing hypertension accounted for a part of our cohort, hypertension caused by bevacizumab was difficult to differentiate from worsening of pre-existing hypertension. Finally, office-based BP measurements might either underestimate or overestimate the true BP values, which could alter classification of the type of hypertension. However, assessing persistence and consistency of BP levels as we did would have mitigated this weakness slightly [43]. Future study should focus on prospective data collection in an attempt to better define best practice and improve data quality.

A potential limitation of landmark analyses is the fact that events, in particular grade > 2 proteinuria, that occur before the landmark are not included in the analysis beyond the landmark. It is also critical to recognize that the 3 month landmark analysis is time specific, and should not be directly generalized to any other time point of interest. To alleviate this issue of landmark analysis, we presented the cumulative incidence of proteinuria both before and after the landmark time point, and simply compared them. Analysis showed that the 3 months cumulative incidence was not significantly different in any of the intergroup comparisons, and therefore was unlikely to have affected the analyses after the landmark. Moreover, we performed sensitivity analyses (data not shown) with a different cutoff point (0 to 6 months), which confirmed the results presented here. In some cases, the use of any other statistical approach, including time-dependent covariate Cox regression might be more appropriate than the landmark analysis approach, but we leave such evaluations for future research [44, 45].

## Conclusions

In this retrospective study of CRC patients treated with bevacizumab, we confirmed that BP status during the first 3 months of treatment influences the risk of subsequent proteinuria. Therefore, timely diagnosis and stricter BP control are recommended for at least the first 3 months after bevacizumab treatment initiation to avoid severe proteinuria. Initiation of aHT agents should be considered when SBP is > 140 mmHg. However, additional studies are warranted to investigate the effectiveness of individual aHT agents on proteinuria and hypertension with bevacizumab treatment, their ideal dosing, and timing and duration of administration. Until this issue is clarified by future research, we suggest that healthcare providers tailor their recommendations on the basis of more established factors, and implement individualized treatment plans based on individual patients’ circumstances through pre-treatment screening and on-treatment monitoring.

### Electronic supplementary material

Below is the link to the electronic supplementary material.

Supplementary Material 1

## Data Availability

The datasets used and/or analyzed during the current study are available from the corresponding author on reasonable request.

## Abbreviations

ACEIs: angiotensin-converting enzyme inhibitors
aHT: antihypertensive
ARBs: angiotensin receptor blockers
BP: blood pressure
CCBs: calcium channel blockers
CI: confidence intervals
CRC: colorectal cancer
CTCAE v5.0: Common Terminology Criteria for Adverse Events version 5.0
DBP: diastolic blood pressures
eGFR: estimated glomerular filtration rate
HR: hazard ratios
RASIs: renin-angiotensin system inhibitors
SBP: systolic blood pressures
UPCR: urine protein-creatinine ratio
VEGF: vascular endothelial growth factor
VEGFRs: vascular endothelial growth factor receptors
