# Supplementary material for: Effect of blood pressure control on the risk of proteinuria during bevacizumab treatment in patients with colorectal cancer: a single-center retrospective cohort study
Source: J Pharm Health Care Sci. 2024 Aug 23;10:51. doi: 10.1186/s40780-024-00372-8 (PMC11342735; doi:10.1186/s40780-024-00372-8)
Supplement: Supplementary file 1 — Supplementary Material 1 [file 40780_2024_372_MOESM1_ESM.docx]

**Supplementary Material**

**Supplementary Table 1**: The use of antihypertensive agents at baseline.

**Supplementary Table 2**: The adding antihypertensive agents during bevacizumab treatment.

**Supplementary Table 1.** Use of antihypertensive agents at baseline

| Agents | Normal SBP (n=170) | High SBP (n=109) |
| --- | --- | --- |
| RASIs | 17 (10) | 31 (28) |
| Olmesartan | 5 (3) | 8 (7) |
| Telmisartan | 3 (2) | 7 (8) |
| Azilsartan | 4 (2) | 5 (3) |
| Candesartan | 3 (2) | 5 (5) |
| Losartan | 1 (1) | 2 (2) |
| Valsartan | 1 (1) | 1 (1) |
| Irbesartan | 0 (0) | 1 (1) |
| Enalapril | 0 (0) | 1 (1) |
| Imidapril | 0 (0) | 1 (1) |
| CCBs | 26 (15) | 30 (28) |
| Amlodipine | 21 (12) | 23 (21) |
| Nifedipine | 5 (3) | 5 (5) |
| Azelnidipine | 0 (0) | 1 (1) |
| Cilnidipine | 0 (0) | 1 (1) |
| Diuretics | 5 (3) | 8 (7) |
| Trichlormethiazide | 3 (2) | 4 (4) |
| Hydrochlorothiazide | 2 (1) | 3 (3) |
| Furosemide | 0 (0) | 1 (1) |
| α/β- or β-blockers | 1 (1) | 2 (2) |
| Bisoprolol | 1 (1) | 1 (1) |
| Carvedilol | 0 (0) | 1 (1) |
| Abbreviations: SBP, systolic blood pressure; RASIs, renin-angiotensin system inhibitors; CCBs, calcium channel blockers. | | |

**Supplementary Table 2.** Antihypertensive agents added during bevacizumab treatment

| Agents | Normal SBP (n=170) | High SBP (n=109) |
| --- | --- | --- |
| RASIs | 24 (14) | 28 (26) |
| Azilsartan | 12 (7) | 15 (14) |
| Olmesartan | 9 (5) | 8 (7) |
| Candesartan | 3 (2) | 5 (5) |
| CCBs | 19 (11) | 22 (20) |
| Amlodipine | 15 (9) | 17 (16) |
| Nifedipine | 3 (2) | 4 (4) |
| Azelnidipine | 0 (0) | 1 (1) |
| Cilnidipine | 1 (1) | 0 (0) |
| Diuretics | 2 (1) | 4 (4) |
| Trichlormethiazide | 1 (1) | 3 (3) |
| Hydrochlorothiazide | 1 (1) | 1 (2) |
| α/β- or β-blockers | 0 (0) | 1 (1) |
| Bisoprolol | 0 (0) | 1 (1) |
| Abbreviations: SBP, systolic blood pressure; RASIs, renin-angiotensin system inhibitors; CCBs, calcium channel blockers. | | |
